# Impact of postoperative blood pressure management on postoperative hemorrhage after resection of intraparenchymal brain tumors

**DOI:** 10.1007/s00701-026-06894-4

**Published:** 2026-04-29

**Authors:** Matthias Demetz, Aleksandrs Krigers, Rodrigo Uribe-Pacheco, Daniel Pinggera, Julia Klingenschmid, Claudius Thomé, Christian F. Freyschlag, Johannes Kerschbaumer

**Affiliations:** Department of Neurosurgery, Medical University of Innsbruck, Anichstr. 35, T6020, Innsbruck, Austria

**Keywords:** Intracranial tumors, Postoperative hemorrhage, Blood pressure management, Neuro-oncology

## Abstract

**Purpose:**

This study aimed to assess the relationship between postoperative blood pressure and other common radiological and epidemiological factors in relation to the incidence of postoperative hemorrhage.

**Methods:**

This retrospective analysis included all patients who underwent surgery between 2016 and 2023 at our institution for an intraparenchymal intracranial tumor. We evaluated blood pressure both intraoperatively and postoperatively for the first 12 postoperative hours. We compared a cohort that experienced postoperative hemorrhage with a cohort that did not require intervention. ROC-analysis were performed to find thresholds for postoperative blood pressure.

**Results:**

453 patients were included. In 35 cases (7.7%), further treatment was necessary due to postoperative hemorrhage. Of these, nine patients required revision surgery, while 26 patients were managed conservatively. Both intra- (*p* < 0.05) and postoperative (*p* < 0.001) blood pressure influenced the risk of postoperative hemorrhage significantly.

We identified a threshold of 140 mmHg of systolic blood pressure to minimize the risk of postoperative hemorrhage.

**Conclusion:**

Maintaining systolic blood pressure below 140 mmHg after surgery seems to be crucial, as patients with postoperative hemorrhage experienced significantly longer durations of elevated systolic pressure (62 min > 140 mmHg and 17 min > 160 mmHg), highlighting its strong influence on hemorrhage risk.

## Introduction

Surgical resection is a cornerstone in the treatment of intracranial gliomas and large, symptomatic brain metastases [6, 7, 10, 11, 17, 27, 33]. However, postoperative complications, such as postoperative hemorrhage and postoperative ischemia, remain a significant concern in managing these patients with a high risk for neurological deterioration as well as reduced survival [9, 16, 30]. Elevated blood pressure during and after surgery has been described as a risk factor for postoperative hemorrhage, which can lead to serious consequences, including prolonged hospital stays, compromised neurological function, and even mortality [4, 28]. On the other hand, inadequate blood pressure control may threaten cerebral perfusion, increasing the risk of ischemia, what might again lead to subsequent neurological deficits [13, 14]. Thus, the challenge lies in optimizing blood pressure management during and after resection for intracranial gliomas and brain metastases to minimize the risks of both hemorrhage and postoperative neurological complications. Achieving this balance necessitates continuous blood pressure monitoring, with some patients requiring transient antihypertensive treatment, typically managed through invasive blood pressure measurements in intermediate or intensive care settings [20, 25, 35].

In recent years, there has been increasing interest in understanding the relationship between blood pressure management and postoperative complications after surgical resection for intracranial neoplasms. Especially in glioma patients, comorbidities and fluctuations in blood pressure can significantly impact postoperative morbidity and outcome [37]. However, standardized blood pressure targets following craniotomy for intracranial neoplasm resection remain vague, highlighting the need for further research [3, 8, 15, 35]. Data on the effects of both intra- and postoperative blood pressure management, as well as other radiological, epidemiological and neuro-oncological factors such as residual tumor volume, gender, preoperative history of hypertension, and patient age on the occurrence of postoperative complications like hemorrhage are limited.

This study aimed to investigate the influence of various preoperative epidemiological, radiological, and neuro-oncological risk factors, along with the impact of both intra- and postoperative blood pressure management, on the development of early postoperative hemorrhage. Additionally, we aimed to identify optimal blood pressure targets after resection of intracranial gliomas and brain metastases to minimize the risk of postoperative hemorrhage.

## Material & methods

In this study we included all adult patients (≥ 18 years at the time of surgery) who underwent elective surgical resection for an intracranial intraparenchymal glioma or brain metastasis at the authors’ institution between January 2016 and November 2023. Epidemiological, clinical and radiological data could be retrieved from the prospective institutional neuro-oncological database and electronic patients’ charts. Initial histological diagnosis was based on the WHO grading system for central nervous system tumors, following the 2016 classification or the 2021 revision [22, 23], depending on the date of diagnosis. The authors reviewed and updated diagnoses originally classified according to the WHO classification of 2016 to align with the 2021 classification where applicable. Patients who underwent unplanned emergency procedures were excluded from this study.

Surgical resection was performed as standard of care at the authors’ institution in all eligible patients according to international guidelines. Patients were seen postoperatively at three- or six-months intervals, depending on the WHO grade and molecular characteristics resected neoplasm. Patients’ general health status and condition were quantified using the Karnofsky Performance Score (KPS) both pre- and postoperatively.

Following surgery, all patients were transferred to the Neurosurgical Intensive Care Unit (ICU) for 18–24 h of monitoring. Each patient was observed for multiple factors including heart rate, neurological status, and blood pressure using an arterial line as standard of care. Hypertension was treated with different antihypertensive medications and pain relief. Postoperative Magnetic resonance imaging (MRI) was performed within 48 h as a standard of care at the authors’ institution for patients with intracranial tumors [12]. In cases of significant hemorrhage, surgical evacuation or prolonged ICU observation including blood pressure management with arterial line were considered as treatment options based on the mass effect and the patient's clinical condition. Postoperative hemorrhage was defined as visible blood in the resection cavity or surrounding brain tissue on postoperative imaging. Treatment-requiring hemorrhage was classified as extensive bleeding that caused significant mass effect associated with new neurological deficits.

Tumor volume was manually assessed both pre- and postoperatively using segmentation in ITK-SNAP software (v.3.8.0 for Mac OS, developed by UPenn and UNC) on T1 contrast-enhanced (CE), native T1, T2, FLAIR (fluid-attenuated inversion recovery), and DWI (diffusion-weighted imaging) sequences [36].

Data evaluation was conducted using IBM SPSS Statistics (Version 27.0 for Mac OS, Armonk, NY: IBM Corp.). For scale variables, T-tests were used if the data followed a normal distribution, with results presented as means and standard deviations (SD). If the data did not follow a normal distribution, the Mann–Whitney U-test was applied, and results were presented as medians with interquartile ranges (IQR). Binomial pairs were compared using the Chi-squared test. Mean progression-free survival (PFS) and overall survival (OS) times were estimated using Kaplan–Meier analysis and compared with the Log-Rank test. Cox regression was employed to determine hazard ratios (HR) for oncological progression or death. An α value of 0.05 was used, and 95% confidence intervals were calculated. Receiver operating characteristic (ROC) curve analysis was performed to evaluate the predictive value of postoperative blood pressure and other relevant factors for postoperative hemorrhage. The area under the curve (AUC) was calculated to assess the discriminatory ability of each variable. To determine the optimal cutoff value for postoperative blood pressure associated with hemorrhage risk, Youden’s Index was applied. This index, defined as the maximum sum of sensitivity and specificity minus one, was used to identify the threshold that best differentiates between patients with and without postoperative hemorrhage.

This study was approved by the Institutional Ethics Committee of the Medical University of Innsbruck (reference number 1291/2024). It was conducted in accordance with the ethical standards set forth in the 1964 Declaration of Helsinki and its subsequent amendments.

## Results

### Patient characteristics

453 patients (252 male, 201 female) with a median age of 59 years (Interquartile range (IQR) 49–69, absolute range 20–85) at surgery were included in this study. 323 patients (71.3%) underwent surgery for an intracranial glioma, while 130 patients (28.7%) underwent resection for brain metastasis. Of the 453 patients included in the study, 415 (91.6%) had tumors located in the supratentorial region, 36 (7.9%) had tumors in the infratentorial region, and 2 (0.4%) patients had tumors in both infra- and supratentorial locations. Regarding tumor laterality, 224 patients (49.4%) had left-sided tumors, 219 (48.3%) had right-sided tumors, and 10 patients (2.2%) had tumors involving both brain hemispheres. Most frequent tumor location was the frontal lobe with 192 patients, followed by the temporal lobe, which was affected in 133 patients. In this series, 152 patients (33.6%) showed a preoperative history of epileptic seizures. Median pre- and postoperative tumor volumes in various MRI sequences as well as extent of resection are shown in Table 1.

**Table 1:** Median preoperative and postoperative tumor volumes as well as mean extent of resection after surgery

MRI sequence	Mean preoperative volume (Standard-Deviation)	Mean postoperative volume (Standard-Deviation)	Mean Extent of Resection (Standard-Deviation)
T1 CE	34.9 cm^3^ (41.5)	1.7 cm^3^ (7.7)	95% (11.2)
T2	38.8 cm^3^ (44.1)	2.1 cm^3^ (8.3)	89% (11.7)

In our series, 381 patients presented with de novo tumors, while 72 had previously undergone treatment. Among the pre-treated patients, 18 had undergone prior surgical resection, 22 had received radiotherapy, and 72 had been treated with systemic chemotherapy.

A total of 147 patients (32.5%) showed a history of arterial hypertension and were on antihypertensive medication prior to surgery.

Median preoperative KPS amounted to 90 (IQR 80–100), indicating a low burden of disease in our patients. The high KPS could be maintained at first follow up (after three months) with a median KPS of 90 (IQR 80–100). We could not find any significant differences for KPS at first follow up between patients with treatment-requiring postoperative hemorrhage and patients without postoperative hemorrhage (*p* > 0.05).

The most frequent histological diagnosis among glioma patients was a glioblastoma (GBM, CNS WHO grade 4) with 231 cases (71.5% of all gliomas). Diffuse glioma CNS WHO grade 2 was diagnosed in 37 patients (11.5%). The diagnosis of an anaplastic glioma CNS WHO grade 3 was confirmed in 55 cases (17.1%). IDH-1 showed a mutation in 28.5% and IDH-1 wildtype in 71.5%.

### Survival

Kaplan–Meier analysis revealed an estimated median OS of 17 months (IQR 8—40).

**Fig. 1:**
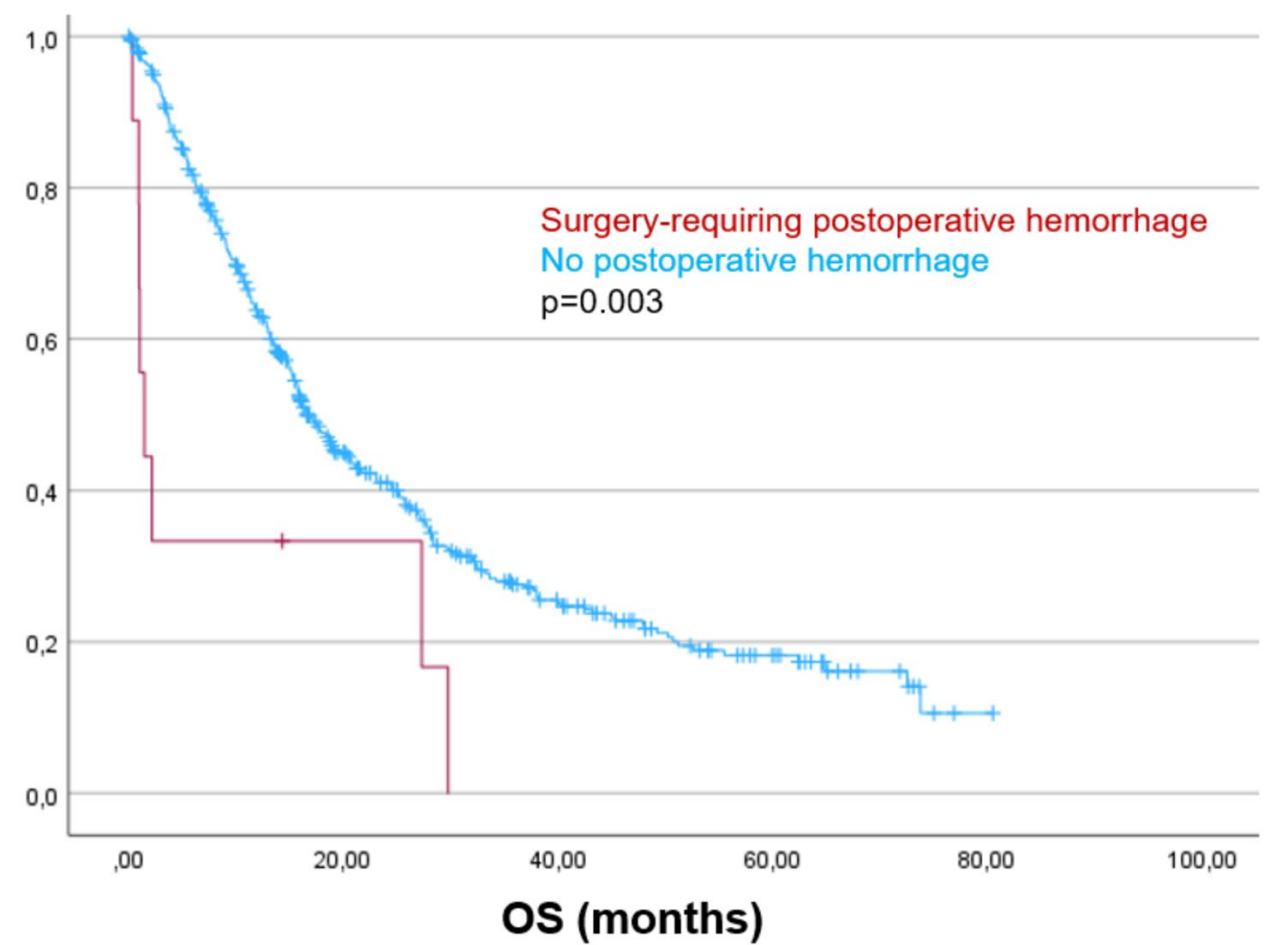
Patients with postoperative hemorrhage with extensive mass effect had significantly worse survival outcome compared to those without postoperative hemorrhage (*p* = 0.003)

**Fig. 2:**
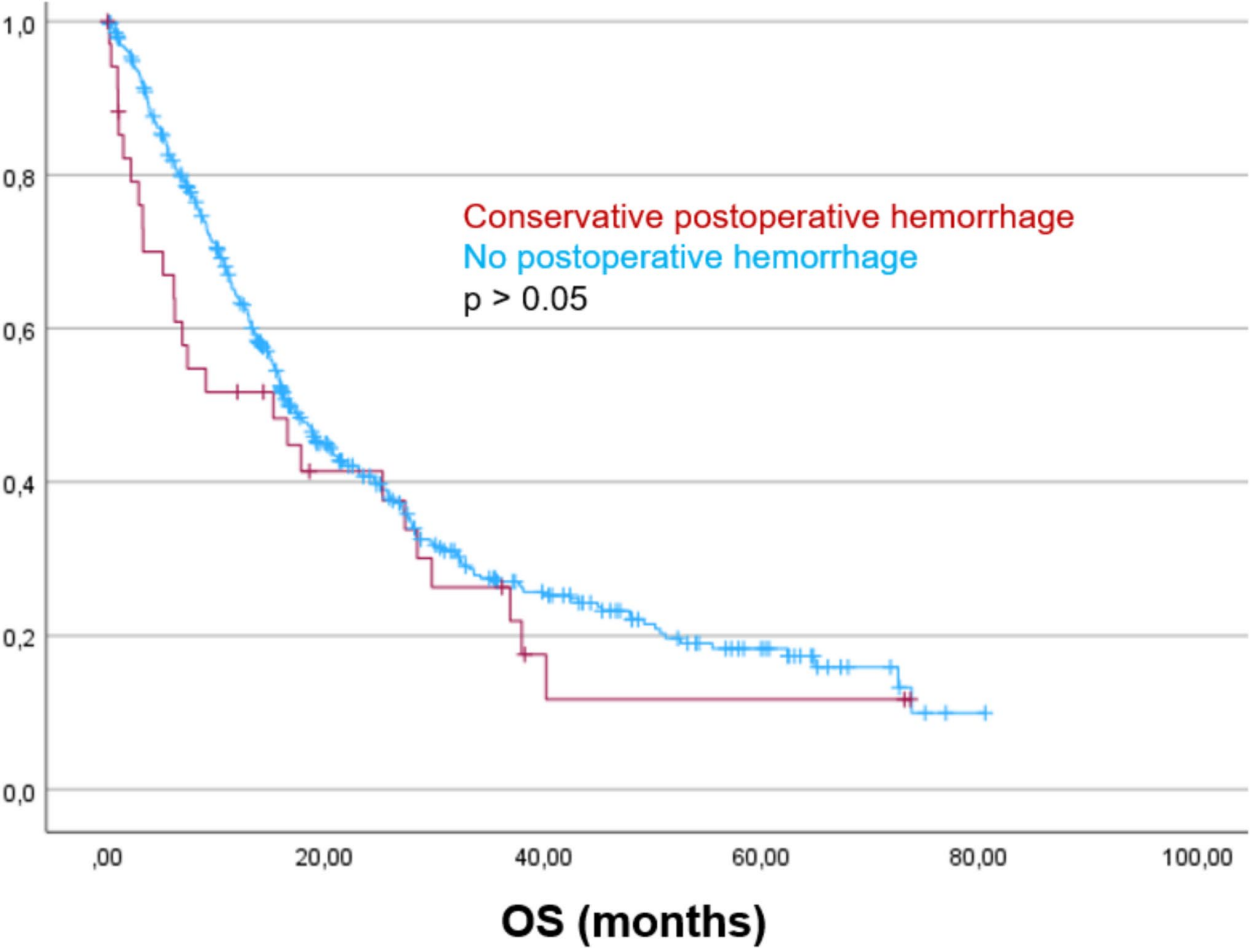
Patients with postoperative hemorrhage that did not require revision surgery showed no significant differences in OS compared to patients without postoperative hemorrhage

Kaplan–Meier survival analysis revealed that patients who required surgical evacuation due to postoperative hemorrhage with extensive mass effect had significantly worse survival outcome compared to those without postoperative hemorrhage (median 1.5 months (IQR 1–27.3) vs median 16.6 months (IQR 8.3—40.2), *p* = 0.003, Fig. 1). However, no significant differences were found in overall survival in between patients with postoperative ischemia and patients without postoperative ischemia median 20.5 months (IQR 8.4—50.7) vs median 16.2 months (IQR 7.9—38), *p* > 0.05. Furthermore, Kaplan–Meier analysis revealed no significant reduced OS between patients with postoperative hemorrhage that did not require revision surgery and patients without postoperative hemorrhage (median 15.2 months (IQR 3.3—36.9) vs median 16.6 months (IQR 8.6—42.5), *p* > 0.05, Fig. 2).

### Postoperative hemorrhage

In the postoperative MRI, a small, non-significant rim of blood was observed in 51 patients (11.3%), all of whom remained asymptomatic and did not require additional intervention. In 35 cases (7.7%) within our patient cohort, further treatment was necessary due to postoperative hemorrhage to prevent potential neurological deterioration. Of these, only nine patients (2% of the cohort) required revision surgery, while the remaining 26 patients were managed conservatively with prolonged ICU observation and intravenous antihypertensive therapy. Small perilesional ischemia was detected on postoperative MRI in 51 cases (11.3%).

In the Cox Regression analysis, OS was significantly reduced in case of postoperative hemorrhage with mass effect that required surgical revision (*p* = 0.004). However, OS was not significantly influenced in cases of postoperative hemorrhage that could be managed conservatively (*p* > 0.05).

**Fig. 3:**
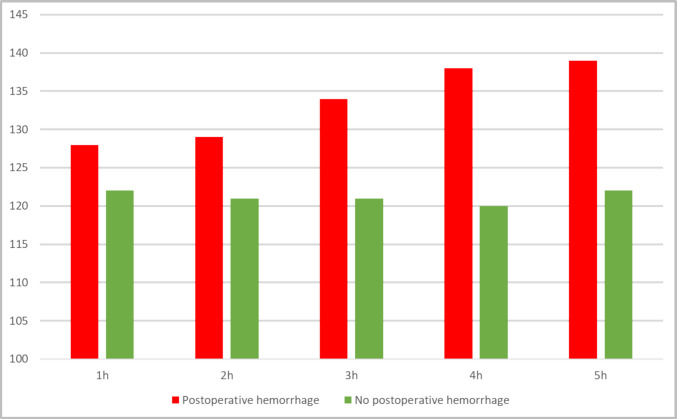
Mean intraoperative systolic blood pressure in mmHg during surgical resection of intracranial intraparenchymal tumors

Differences in mean intraoperative systolic blood pressure between patients with treatment-requiring postoperative hemorrhage and no postoperative hemorrhage can be found in Fig. 3.

Patients who required any treatment for the postoperative hemorrhage had a mean duration of 62 min (SD ± 48) with systolic blood pressure above 140 mmHg during surgery, compared to a mean duration of 17 min (SD ± 16) in patients without postoperative hemorrhage.

**Fig. 4:**
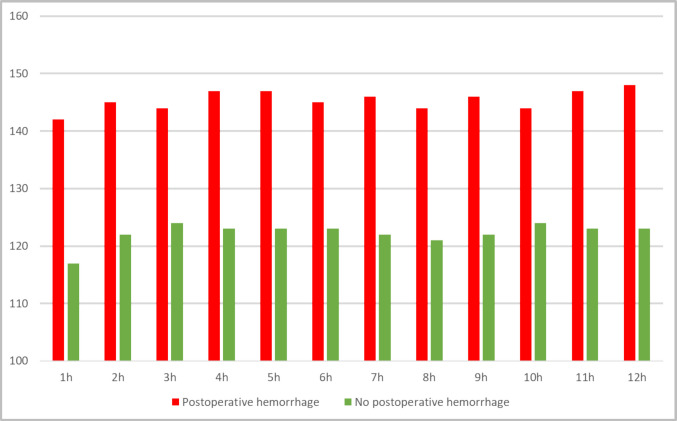
Mean postoperative systolic blood pressure in mmHg after surgical resection of intracranial intraparenchymal tumors

Differences in mean postoperative systolic blood pressure between patients with treatment-requiring postoperative hemorrhage and no postoperative hemorrhage are shown in Fig. 4.

**Fig. 5:**
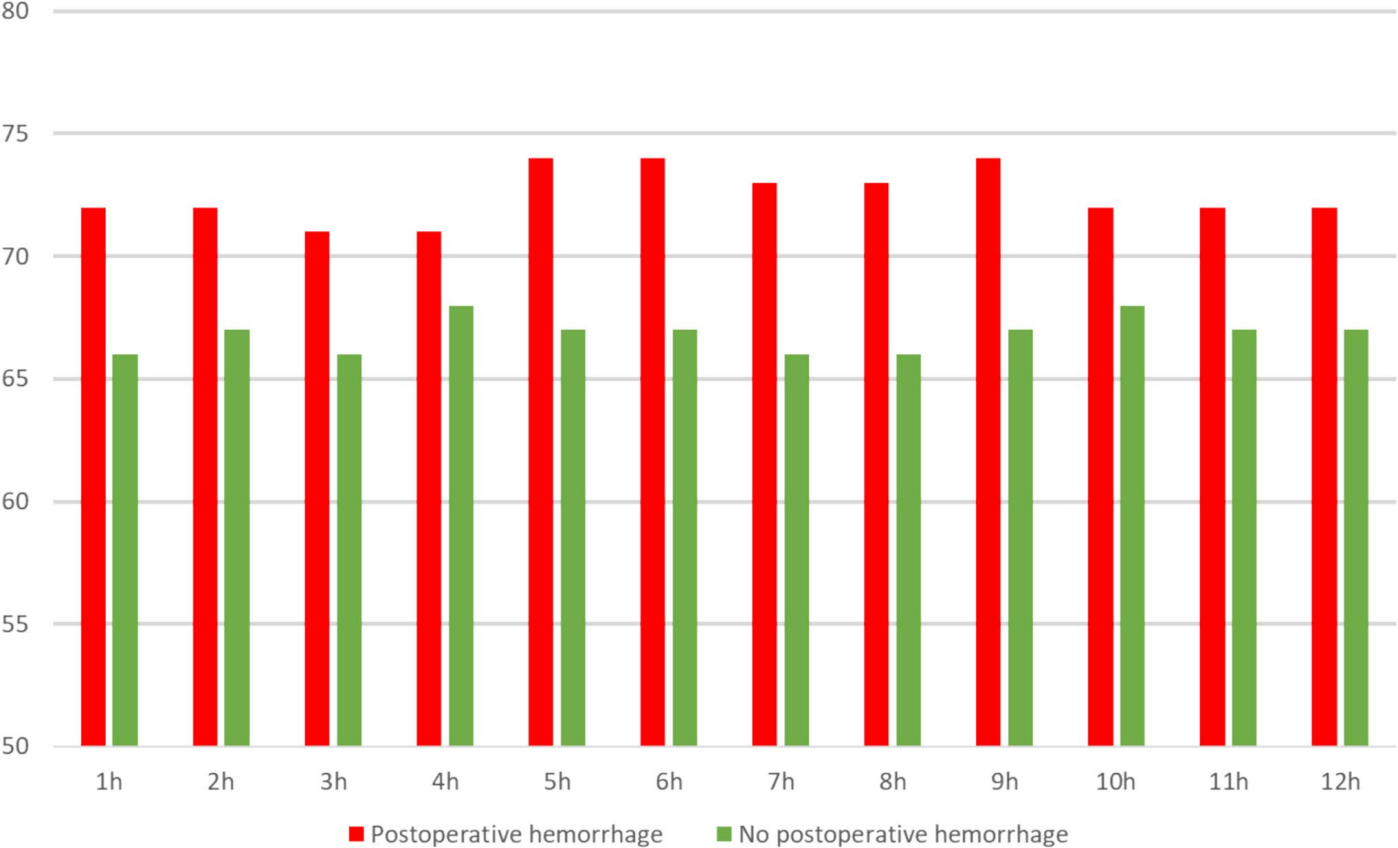
Mean intraoperative heart rate in bpm after surgical resection of intracranial intraparenchymal tumors

Postoperative heart rate in beats per minute (bpm) was assessed to objectively measure the stress levels in our patients. The differences in mean postoperative heart rate per hour between patients with treatment-requiring postoperative hemorrhage and no postoperative hemorrhage are shown in Fig. 5. No significant difference was found in mean intraoperative heart rate between patients with postoperative hemorrhage and those without hemorrhage (*p* > 0.05).

The risk of postoperative treatment-requiring hemorrhage was significantly higher among patients with several specific factors: male gender (*p* = 0.008), preoperative history of hypertension (*p* = 0.039), larger preoperative tumor volumes as measured on T1 CE imaging (*p* = 0.014), and a higher residual tumor volume on T1 CE imaging (*p* = 0.031). We could not find any significant differences for age, WHO grade and molecular characteristics in glioma patients, tumor location, preoperative epileptic seizures and previous treatment (all *p* > 0.05).

ROC analysis for postoperative hemorrhage requiring further surgical or conservative intervention revealed significant differences in systolic blood pressure between the two cohorts during all of the first 12 postoperative hours: 1 (*p* < 0.001), 2 (*p* < 0.001), 3 (*p* < 0.001), 4 (*p* < 0.001), 5 (*p* < 0.001), 6 (*p* < 0.001), 7 (*p* < 0.001), 8 (*p* < 0.001), 9 (*p* < 0.001), 10 (*p* < 0.001), 11 (*p* < 0.001), and 12 (*p* < 0.001). For intraoperative systolic blood pressure, significant differences were found during each of the five hours: 1 (*p* = 0.005), 2 (*p* = 0.032), 3 (*p* = 0.018), 4 (*p* = 0.023), and 5 (*p* = 0.001). The Youden’s index results are presented in Table 2.

**Table 2:** Youden’s index showed intraoperative systolic blood pressure results of approximately 120–135 mmHg

Hour	Systolic blood pressure (mmHg)
Intraoperative 1	123
Intraoperative 2	123
Intraoperative 3	128
Intraoperative 4	133
Intraoperative 5	128
Postoperative 1	140
Postoperative 2	140
Postoperative 3	139
Postoperative 4	140
Postoperative 5	142
Postoperative 6	141
Postoperative 7	143
Postoperative 8	140
Postoperative 9	141
Postoperative 10	139
Postoperative 11	140
Postoperative 12	141

For the postoperative course, systolic blood pressure results of approximately 140 mmHg could be found

We furthermore identified significant differences in the ROC analysis for time spent above certain systolic blood pressure thresholds. Time spent with systolic blood pressure over 140 mmHg was significantly associated with postoperative treatment requiring hemorrhage (*p* = 0.006), as was time over 160 mmHg (*p* < 0.001) and over 180 mmHg (*p* < 0.001). Youden’s index was 115 min for time over 140 mmHg, 18 min for over 160 mmHg and 2 min for the time spent over 180 mmHg.

We found no significant difference in postoperative blood pressure between patients with postoperative ischemia and those without postoperative ischemia (p > 0.05), indicating that the blood pressure level post-surgery was not associated with the development of ischemic events.

## Discussion

In this retrospective trial, we identified a critical threshold of 140 mmHg for postoperative systolic blood pressure, which should be applied to minimize the risk of hemorrhage. In our cohort, we reported early postoperative hemorrhage in 7.7% of our patients following surgery for intracranial tumors, with 2% undergoing revision surgery, supporting the existing literature on the risk of adverse events after elective craniotomy [5, 21, 34]. Our study highlights that postoperative hemorrhage remains a serious complication possibly influencing OS, with significantly higher risk observed in patients with pre-existing cardiovascular risk factors, such as male gender and a history of hypertension. Patients who developed postoperative hemorrhage demonstrated significantly higher systolic blood pressure levels during and after surgery. Importantly, we found no significant differences in postoperative ischemia between patients with or without hemorrhage, suggesting that maintaining lower systolic blood pressure did not increase the risk of ischemic events.

Postoperative hemorrhage in intracranial neoplasms can be disastrous for patients, significantly impacting both morbidity and mortality. Preventing such complications is crucial to improving patient outcome [2, 28]. Previous literature already suggested that systolic blood pressure might play a critical role in the development of postoperative hemorrhage; however, standardized blood pressure goals in this context remain poorly defined [4]. While maintaining careful blood pressure control appears vital, there is no clear consensus on the optimal thresholds. Additionally, previous literature has largely focused on patients requiring surgical revisions, potentially overlooking a broader spectrum of patients with postoperative hemorrhage who did not undergo surgery but may still experience adverse outcome [18, 19, 32]. This highlights the need for a more comprehensive approach to managing and preventing postoperative hemorrhage.

Our study included a large cohort of 453 patients with various types of intracranial tumors, including GBM, lower-grade gliomas, and brain metastases. Importantly, we found no significant differences in the incidence of postoperative hemorrhage between the different tumor types. This finding suggests that blood pressure management plays a crucial role in the postoperative care of all patients with intraparenchymal neoplasms, regardless of the specific tumor type.

Our proactive approach to early intervention in cases of postoperative hemorrhage focused on maintaining patients' high preoperative performance status and preventing neurological decline. This approach proved effective, as our findings showed no significant difference in the KPS after three months between patients who experienced postoperative hemorrhage and those who did not. Despite the occurrence of hemorrhage, timely treatment appeared to mitigate its potential impact on functional outcome. However, overall survival was significantly worse in patients who required surgical revision due to mass effect from the hemorrhage. This suggests that the reduced survival may be linked to the harmful impact of mass effect due to large postoperative hemorrhage, highlighting the critical need to prevent postoperative hemorrhage in order to improve long-term patient outcome [2].

Considering our findings, postoperative hemorrhage appears to be closely linked to elevated blood pressure levels. Patients who experienced postoperative hemorrhage had significantly higher blood pressure both intra- and postoperatively compared to those without hemorrhage. Higher systolic blood pressure might be particularly detrimental, potentially causing damage to fragile blood vessels and the brain tissue around the resection cavity, as previously suggested in the literature [1, 31]. Additionally, these patients also exhibited elevated heart rates, suggesting higher postoperative stress levels that may contribute to the risk of hemorrhage. This highlights the importance of managing these stressors through the use of antihypertensive and pain medications. Moreover, patients with a history of hypertension seem to be at greater risk for postoperative hemorrhage, emphasizing the need for heightened vigilance and tailored blood pressure management in this subgroup. Considering our findings, vulnerable patients at higher risk of postoperative hemorrhage should be closely monitored and managed in an ICU after surgery to ensure optimal blood pressure control and early intervention if complications arise.

Postoperative hemorrhage can lead to significant morbidity and mortality in patients undergoing surgery for intracranial intraparenchymal tumors, as previously shown [19, 26]. Our data further substantiate the relevance of this complication by demonstrating a significant impact on overall survival in cases requiring surgical revision due to mass effect. The imperative to avoid postoperative hemorrhage is grounded not only in preserving immediate postoperative outcome but also in safeguarding long-term survival, as highlighted in the existing literature. Based on our findings and building upon previous research by Young et al. [15, 35], we support the establishment of a rigid systolic blood pressure threshold of 140 mmHg during the early postoperative period. Furthermore, we suggest considering an even lower intraoperative blood pressure goal of approximately 130 mmHg to potentially minimize the risk of postoperative hemorrhage in patients undergoing surgery for intracranial intraparenchymal tumors.

However, the proposed blood pressure limit of 140 mmHg should be applied judiciously, taking into account individual patient characteristics. The control group without postoperative hemorrhage did not exhibit higher incidences of postoperative ischemia, suggesting that adhering to this threshold did not increase the risk of ischemic events. Our data further indicate that a brief period exceeding 140 mmHg may not be fatal. This is underlined by a Youden’s index of 115 min. However, it is crucial to consider that the risk of hemorrhage increases with higher blood pressure, and thus, thus the duration increased blood pressure should be minimized. Specifically, a short duration of 18 min of over 160 mmHg and an even a shorter duration of just 2 min above 180 mmHg significantly elevates the risk for bleeding, underscoring the need for careful and individualized blood pressure management during the postoperative period.

Tumor volumes influenced the risk of postoperative hemorrhage in our study. We found that patients with larger preoperative tumor volumes and larger residual tumor volumes after surgery were at increased risk for hemorrhage. The elevated risk for hemorrhage in patients with larger preoperative tumor volumes may be attributed to the greater likelihood of residual tumor tissue after resection, as supported by previous literature [18]. Residual tumor volumes containing highly vascularized tissue, particularly in highly malignant neoplasms such as GBM or brain metastases, might explain their elevated risk [24, 29]. Blood pressure should be closely monitored in patients with suspected residual tumor volume to mitigate the risk of postoperative hemorrhage.

While our study offers valuable insights into the complexities of blood pressure managements after surgical resection of intracranial intraparenchymal neoplasms, several limitations must be acknowledged. Future research involving diverse prospective randomized multicentric cohorts is needed to validate and broaden the applicability of our findings. Our focus was primarily on early hemorrhagic events and blood pressure values within a specific timeframe, limiting our ability to draw comprehensive conclusions about long-term blood pressure management, particularly beyond the immediate postoperative period. Additionally, the decision for surgical revision was made on a case-by-case basis, considering factors such as hemorrhage location, volume, mass effect, and clinical presentation, which may limit the generalizability of our findings.

## Conclusion

Postoperative hemorrhage is a potential complication in patients undergoing surgery for intracranial intraparenchymal neoplasms. To minimize this risk, treating physicians should aim to maintain the systolic blood pressure below 140 mmHg during the postoperative period. Special attention should be given to patients with risk factors such as a history of hypertension and suspected residual tumor to mitigate the risk of postoperative hemorrhage.

## Data Availability

The data supporting this study are available from the corresponding author upon reasonable request.

## References

[1] Abaziou T, Tincres F, Mrozek S, Brauge D, Marhar F, Delamarre L et al (2020) Incidence and predicting factors of perioperative complications during monitored anesthesia care for awake craniotomy. J Clin Anesth 64:109811. 10.1016/j.jclinane.2020.109811 32320919 10.1016/j.jclinane.2020.109811

[2] Ahmadipour Y, Kaur M, Pierscianek D, Gembruch O, Oppong MD, Mueller O et al (2019) Association of surgical resection, disability, and survival in patients with glioblastoma. J Neurol Surg A Cent Eur Neurosurg 80:262–268. 10.1055/s-0039-1685170 30965373 10.1055/s-0039-1685170

[3] Anderson CS, Heeley E, Huang Y, Wang J, Stapf C, Delcourt C et al (2013) Rapid blood-pressure lowering in patients with acute intracerebral hemorrhage. N Engl J Med 368:2355–2365. 10.1056/NEJMoa1214609 23713578 10.1056/NEJMoa1214609

[4] Basali A, Mascha EJ, Kalfas I, Schubert A (2000) Relation between perioperative hypertension and intracranial hemorrhage after craniotomy. Anesthesiology 93:48–54. 10.1097/00000542-200007000-00012 10861145 10.1097/00000542-200007000-00012

[5] Bette S, Wiestler B, Wiedenmann F, Kaesmacher J, Bretschneider M, Barz M et al (2017) Safe brain tumor resection does not depend on surgery alone - role of hemodynamics. Sci Rep 7:5585. 10.1038/s41598-017-05767-2 28717226 10.1038/s41598-017-05767-2 PMC5514064

[6] Brown TJ, Brennan MC, Li M, Church EW, Brandmeir NJ, Rakszawski KL et al (2016) Association of the extent of resection with survival in glioblastoma. JAMA Oncol 2:1460. 10.1001/jamaoncol.2016.1373 27310651 10.1001/jamaoncol.2016.1373 PMC6438173

[7] Demetz M, Krigers A, Moser P, Kerschbaumer J, Thomé C, Freyschlag CF (2023) Same but different. Incidental and symptomatic lower grade gliomas show differences in molecular features and survival. J Neurooncol 162:397–405. 10.1007/s11060-023-04301-x 37043120 10.1007/s11060-023-04301-x PMC10167120

[8] Demetz M, Krigers A, Uribe-Pacheco R, Pinggera D, Klingenschmid J, Thomé C et al (2024) The role of postoperative blood pressure management in early postoperative hemorrhage in awake craniotomy glioma patients. Neurosurg Rev 47:452. 10.1007/s10143-024-02661-0 39168945 10.1007/s10143-024-02661-0 PMC11339099

[9] Di Carlo DT, Cagnazzo F, Anania Y, Duffau H, Benedetto N, Morganti R et al (2020) Post-operative morbidity ensuing surgery for insular gliomas: a systematic review and meta-analysis. Neurosurg Rev 43:987–997. 10.1007/s10143-019-01113-4 31098791 10.1007/s10143-019-01113-4

[10] Dono A, Zhu P, Takayasu T, Arevalo O, Riascos R, Tandon N et al (2024) Extent of resection thresholds in molecular subgroups of newly diagnosed Isocitrate Dehydrogenase–wildtype glioblastoma. Neurosurgery 95:932–940. 10.1227/neu.0000000000002964 38687046 10.1227/neu.0000000000002964 PMC12245224

[11] Felsberg J, Rapp M, Loeser S, Fimmers R, Stummer W, Goeppert M et al (2009) Prognostic significance of molecular markers and extent of resection in primary glioblastoma patients. Clin Cancer Res 15:6683–6693. 10.1158/1078-0432.CCR-08-2801 19861461 10.1158/1078-0432.CCR-08-2801

[12] Freyschlag CF, Krieg SM, Kerschbaumer J, Pinggera D, Forster M-T, Cordier D et al (2018) Imaging practice in low-grade gliomas among European specialized centers and proposal for a minimum core of imaging. J Neurooncol 139:699–711. 10.1007/s11060-018-2916-3 29992433 10.1007/s11060-018-2916-3 PMC6132968

[13] Gempt J, Krieg SM, Hüttinger S, Buchmann N, Ryang Y-M, Shiban E et al (2013) Postoperative ischemic changes after glioma resection identified by diffusion-weighted magnetic resonance imaging and their association with intraoperative motor evoked potentials. J Neurosurg 119:829–836. 10.3171/2013.5.JNS121981 23829818 10.3171/2013.5.JNS121981

[14] Gempt J, Förschler A, Buchmann N, Pape H, Ryang Y-M, Krieg SM et al (2013) Postoperative ischemic changes following resection of newly diagnosed and recurrent gliomas and their clinical relevance. J Neurosurg 118:801–808. 10.3171/2012.12.JNS12125 23373806 10.3171/2012.12.JNS12125

[15] Hanak BW, Walcott BP, Nahed BV, Muzikansky A, Mian MK, Kimberly WT et al (2014) Postoperative intensive care unit requirements after elective craniotomy. World Neurosurg 81:165–172. 10.1016/j.wneu.2012.11.068 23182731 10.1016/j.wneu.2012.11.068 PMC3596491

[16] Karsy M, Yoon N, Boettcher L, Jensen R, Shah L, MacDonald J et al (2018) Surgical treatment of glioblastoma in the elderly: the impact of complications. J Neurooncol 138:123–132. 10.1007/s11060-018-2777-9 29392589 10.1007/s11060-018-2777-9

[17] Kerschbaumer J, Demetz M, Krigers A, Pinggera D, Spinello A, Thomé C et al (2023) Mind the gap-the use of sodium fluoresceine for resection of brain metastases to improve the resection rate. Acta Neurochir (Wien) 165:225–230. 10.1007/s00701-022-05417-1 36369398 10.1007/s00701-022-05417-1 PMC9840582

[18] Laurent D, Freedman R, Cope L, Sacks P, Abbatematteo J, Kubilis P et al (2020) Impact of extent of resection on incidence of postoperative complications in patients with glioblastoma. Neurosurgery 86:625–630. 10.1093/neuros/nyz313 31342060 10.1093/neuros/nyz313 PMC7594111

[19] Liu W, Qdaisat A, Yeung J, Lopez G, Weinberg J, Zhou S et al (2019) The association between common clinical characteristics and postoperative morbidity and overall survival in patients with glioblastoma. Oncologist 24:529–536. 10.1634/theoncologist.2018-0056 30049883 10.1634/theoncologist.2018-0056 PMC6459250

[20] Lombardi G, Barresi V, Castellano A, Tabouret E, Pasqualetti F, Salvalaggio A et al (2020) Clinical management of diffuse low-grade gliomas. Cancers (Basel) 12. 10.3390/cancers12103008 10.3390/cancers12103008 PMC7603014 33081358

[21] Lonjaret L, Guyonnet M, Berard E, Vironneau M, Peres F, Sacrista S et al (2017) Postoperative complications after craniotomy for brain tumor surgery. Anaesth Crit Care Pain Med 36:213–218. 10.1016/j.accpm.2016.06.012 27717899 10.1016/j.accpm.2016.06.012

[22] Louis DN, Perry A, Reifenberger G, von Deimling A, Figarella-Branger D, Cavenee WK et al (2016) The 2016 World Health Organization classification of tumors of the central nervous system: a summary. Acta Neuropathol 131:803–820. 10.1007/s00401-016-1545-1 27157931 10.1007/s00401-016-1545-1

[23] Louis DN, Perry A, Wesseling P, Brat DJ, Cree IA, Figarella-Branger D et al (2021) The 2021 WHO classification of tumors of the central nervous system: a summary. Neuro Oncol 23:1231–1251. 10.1093/neuonc/noab106 34185076 10.1093/neuonc/noab106 PMC8328013

[24] Mosteiro A, Pedrosa L, Ferrés A, Diao D, Sierra À, González JJ (2022) The vascular microenvironment in glioblastoma: a comprehensive review. Biomedicines 10:1285. 10.3390/biomedicines10061285 35740307 10.3390/biomedicines10061285 PMC9219822

[25] Perez CA, Stutzman S, Jansen T, Perera A, Jannusch S, Atem F et al (2020) Elevated blood pressure after craniotomy: a prospective observational study. J Crit Care 60:235–240. 10.1016/j.jcrc.2020.08.013 32942161 10.1016/j.jcrc.2020.08.013

[26] Saunders CN, Cornish AJ, Kinnersley B, Law PJ, Claus EB, Il’yasova D et al (2019) Lack of association between modifiable exposures and glioma risk: a Mendelian randomisation analysis. Neuro Oncol. 10.1093/neuonc/noz209 10.1093/neuonc/noz209 PMC7442418 31665421

[27] Schödel P, Jünger ST, Wittersheim M, Reinhardt HC, Schmidt N, Goldbrunner R et al (2020) Surgical resection of symptomatic brain metastases improves the clinical status and facilitates further treatment. Cancer Med 9:7503–7510. 10.1002/cam4.3402 32858763 10.1002/cam4.3402 PMC7571801

[28] Seifman MA, Lewis PM, Rosenfeld JV, Hwang PYK (2011) Postoperative intracranial haemorrhage: a review. Neurosurg Rev 34:393–407. 10.1007/s10143-010-0304-3 21246389 10.1007/s10143-010-0304-3

[29] Tatla AS, Justin AW, Watts C, Markaki AE (2021) A vascularized tumoroid model for human glioblastoma angiogenesis. Sci Rep 11:19550. 10.1038/s41598-021-98911-y 34599235 10.1038/s41598-021-98911-y PMC8486855

[30] Tuohy K, Ba DM, Bhanja D, Leslie D, Liu G, Mansouri A (2023) Early costs and complications of first-line low-grade glioma treatment using a large national database: limitations and future perspectives. Front Surg. 10.3389/fsurg.2023.1001741 36816005 10.3389/fsurg.2023.1001741 PMC9935584

[31] Wang C, Niu X, Ren Y, Lan Z, Zhang Y (2019) Risk factors for postoperative intracranial hemorrhage after resection of intracranial tumor in 2259 consecutive patients. World Neurosurg 129:e663–e668. 10.1016/j.wneu.2019.05.239 31176060 10.1016/j.wneu.2019.05.239

[32] Wilhelmy F, Gaier M, Planitzer U, Kasper J, Prasse G, Frydrychowicz C et al (2023) Venous thromboembolism and intracranial hemorrhage in patients undergoing glioblastoma surgery. Sci Rep 13:21679. 10.1038/s41598-023-48542-2 38066037 10.1038/s41598-023-48542-2 PMC10709630

[33] Winther RR, Hjermstad MJ, Skovlund E, Aass N, Helseth E, Kaasa S et al (2022) Surgery for brain metastases-impact of the extent of resection. Acta Neurochir (Wien) 164:2773–2780. 10.1007/s00701-021-05104-7 35080651 10.1007/s00701-021-05104-7 PMC9519668

[34] Yang Y-C, Chen Y-S, Liao W-C, Yin C-H, Lin Y-S, Chen M-W et al (2023) Significant perioperative parameters affecting postoperative complications within 30 days following craniotomy for primary malignant brain tumors. Perioper Med 12:54. 10.1186/s13741-023-00343-x 10.1186/s13741-023-00343-x PMC10594926 37872604

[35] Young JS, Morshed RA, Hervey-Jumper SL, Berger MS (2023) The surgical management of diffuse gliomas: current state of neurosurgical management and future directions. Neuro Oncol 25:2117–2133. 10.1093/neuonc/noad133 37499054 10.1093/neuonc/noad133 PMC10708937

[36] Yushkevich PA, Piven J, Hazlett HC, Smith RG, Ho S, Gee JC et al (2006) User-guided 3D active contour segmentation of anatomical structures: significantly improved efficiency and reliability. Neuroimage 31:1116–1128. 10.1016/j.neuroimage.2006.01.015 16545965 10.1016/j.neuroimage.2006.01.015

[37] Zhang Y, Ji P, Wang S, Qin H, Cai Q (2022) Early unplanned reoperation after glioma craniotomy: incidence, predictor and process improvement. Front Oncol. 10.3389/fonc.2022.898873 35600362 10.3389/fonc.2022.898873 PMC9121807

